# Inter-rater reliability of MRI Neck Imaging Reporting and Data System (NI-RADS) in the follow-up of oropharyngeal squamous cell carcinoma

**DOI:** 10.1007/s11547-026-02206-z

**Published:** 2026-03-30

**Authors:** Andrea Falzone, Marco Parillo, Marinella Neri, Alessandro Marinetti, Matteo Zanini, Francesco Sella, Carlo Cosimo Quattrocchi

**Affiliations:** 1 Radiology, Multizonal Unit of Rovereto and Arco, ASUIT Provincia Autonoma Di Trento, Trento, Italy; 2 University of Verona, Verona, Italy; 3 Centre for Medical Sciences - CISMed, University of Trento, Trento, Italy

**Keywords:** Head and Neck neoplasms, Squamous cell carcinoma, Magnetic resonance imaging, Diagnostic imaging, Reproducibility of results, Practice guideline

## Abstract

**Purpose:**

To assess the agreement of Neck Imaging Reporting and Data System (NI-RADS) using magnetic resonance imaging (MRI) among readers with different experience in the evaluation of oropharyngeal squamous cell carcinoma (OPSCC) patients.

**Material and methods:**

We conducted an observational retrospective study and collected post-treatment follow-up MRIs in patients treated for OPSCC. Each scan was scored according to NI-RADS by 1 general radiologist, 2 radiology residents, and 2 seasoned radiologists. Percentage of agreement (POA) and kappa values (κ) were calculated for the assignment of NI-RADS and its individual MRI features (lymph node, primary tumor size, primary site signal on T2-weighted, contrast-enhanced and diffusion-weighted images). Inter-reader agreement was calculated for all post-treatment MRIs and separately for the first post-treatment MRI (using pre-treatment MRI as reference) and subsequent follow-ups.

**Results:**

Ninety-one patients were included (a total of 218 MRIs per rater). The agreement among all readers for NI-RADS (κ = 0.53, POA = 89%) and for each individual MRI feature (κ = 0.42–0.52, POA = 84–93%) assessment was moderate. Lower reliability emerged between the expert radiologist and the radiologists not specialized in head and neck imaging in the first follow-up MRI scan for both primary site contrast enhancement (κ = 0.38-0.41, POA = 72%-88%) and lymph node (κ = 0.25-0.36, POA = 77%-90%) assessment.

**Conclusion:**

MRI NI-RADS showed moderate inter-rater agreement in OPSCC patients, with greater interpretative challenges in the evaluation of the first post-treatment MRI. Regular application of the NI-RADS in clinical settings may help enhance consistency and reliability in imaging evaluations.

**Supplementary Information:**

The online version contains supplementary material available at 10.1007/s11547-026-02206-z.

## Introduction

Oropharyngeal squamous cell carcinoma (OPSCC) has shown a rising incidence across developed nations [1]. In the United States, for instance, its incidence increased by 22% between 1999 and 2006, climbing from 1.53 to 1.87 cases per 100,000 individuals [2]. European data from 2000–2007 show an annual crude incidence of 3.3/100,000 for OPSCC, with a 5-year relative survival rate of 41% [3]. Treating early OPSCC often involves either radical radiotherapy or transoral surgery with neck dissection. Standard care for locally advanced OPSCC includes surgery plus subsequent chemoradiotherapy or primary chemoradiotherapy alone [2, 4].

Complete assessment and staging of OPSCC invariably require cross-sectional imaging. For primary tumor staging, contrast-enhanced magnetic resonance imaging (CE-MRI) is the optimal technique, especially for evaluating soft tissue extension in areas like the tongue base and/or body [5, 6]. For the follow-up of individuals with node-positive disease after completing chemoradiotherapy, fluorodeoxyglucose-positron emission tomography (FDG-PET) is the recommended imaging modality at the 3-month interval to ascertain the necessity of subsequent neck dissection [4, 7, 8]. However, CE-MRI should be employed in the event of symptomatic presentation or the identification of abnormalities during clinical assessment [4].

Recognizing that diverse reporting methods hindered the clear interpretation of imaging for head and neck cancer patients [9], the American College of Radiology (ACR) created the Neck Imaging Reporting and Data System (NI-RADS) to standardize reports and enhance diagnostic clarity [10, 11]. While initially created for follow-up with contrast-enhanced computed tomography (CE-CT) and FDG-PET, NI-RADS has recently been adjusted for application with CE-MRI [12]. By providing a uniform reporting vocabulary, NI-RADS assists radiologists in understanding the challenging post-treatment imaging environment, often featuring anatomical alterations from reconstruction and radiation-induced tissue changes. This standardized language also facilitates communication with referring physicians and supports well-reasoned choices regarding future patient management [13]. In addition to establishing its diagnostic and prognostic utility [14, 15], the routine application of NI-RADS, much like other RADS frameworks [16–18], necessitates further validation through inter-observer agreement studies. Suboptimal interpretative concordance, reflected in low inter-rater agreement, may compromise the clinical value of the system. With this study, we aim to expand the data regarding the reliability of MRI NI-RADS by analyzing a large cohort of patients with a common head and neck cancer (i.e., OPSCC) and investigating the role of experience in assigning the various features constituting the NI-RADS.

## Material and methods

### Study design

This retrospective observational study received approval from the institutional ethics committee (ID code: 2024-087ESA) and was conducted in accordance with the principles outlined in the 2013 Declaration of Helsinki. Given the study’s retrospective design and reliance solely on previously collected, anonymized data, the requirement for informed consent was waived.

Patients were selected from those discussed at the multidisciplinary head-and-neck tumor boards between March 1, 2010 and June 30, 2024; this screening identified 324 patients with oropharyngeal cancer. Inclusion criteria were: histologically confirmed OPSCC; availability of ≥ 2 consecutive post-treatment head-and-neck CE-MRI examinations; and adequate clinical documentation of the treatment modality and the end-of-treatment date. Exclusion criteria were: any prior head-and-neck cancer treatment before the index OPSCC therapy; absence of a pre-treatment CE-MRI (patients staged only with CE-CT); pre-treatment CE-MRI not retrievable because it had been performed at outside institutions; follow-up imaging performed using CE-CT or FDG-PET rather than CE-MRI; MRI examinations lacking post-contrast sequences; and non-diagnostic CE-MRI quality due to motion and/or dental prosthesis artifacts affecting more than one sequence, thereby precluding NI-RADS assignment.

### CE-MRI scans

CE-MRI scans of the head and neck were performed using an institutional protocol [19] on 1.5-Tesla scanners (Optima MR450w by GE HealthCare and Magnetom Aera by Siemens) across six hospitals. The majority of these examinations were carried out at two main centers, accounting for 55% and 24% of the total scans, respectively. Each CE-MRI examination included unenhanced turbo spin-echo images (axial and coronal T2-weighted imaging, axial T1-weighted imaging), axial diffusion-weighted imaging (DWI), and post-contrast 3D gradient echo T1-weighted with fat saturation images. In 10% of the CE-MRI scans, DWI was not available, while 1% of the T2-weighted images were affected by artifacts that impaired image quality. All other sequences included in each CE-MRI examination were fully accessible to the readers for review.

### Image assessment

Five radiologists with varying levels of experience independently reviewed the imaging studies between October 1, 2024, and April 30, 2025. The group consisted of two seasoned head and neck radiologists with 23 and 22 years of experience (Readers A and B), a general radiologist with 21 years of experience (Reader C), and two fourth-year radiology residents (Readers D and E).

**Fig. 1:**
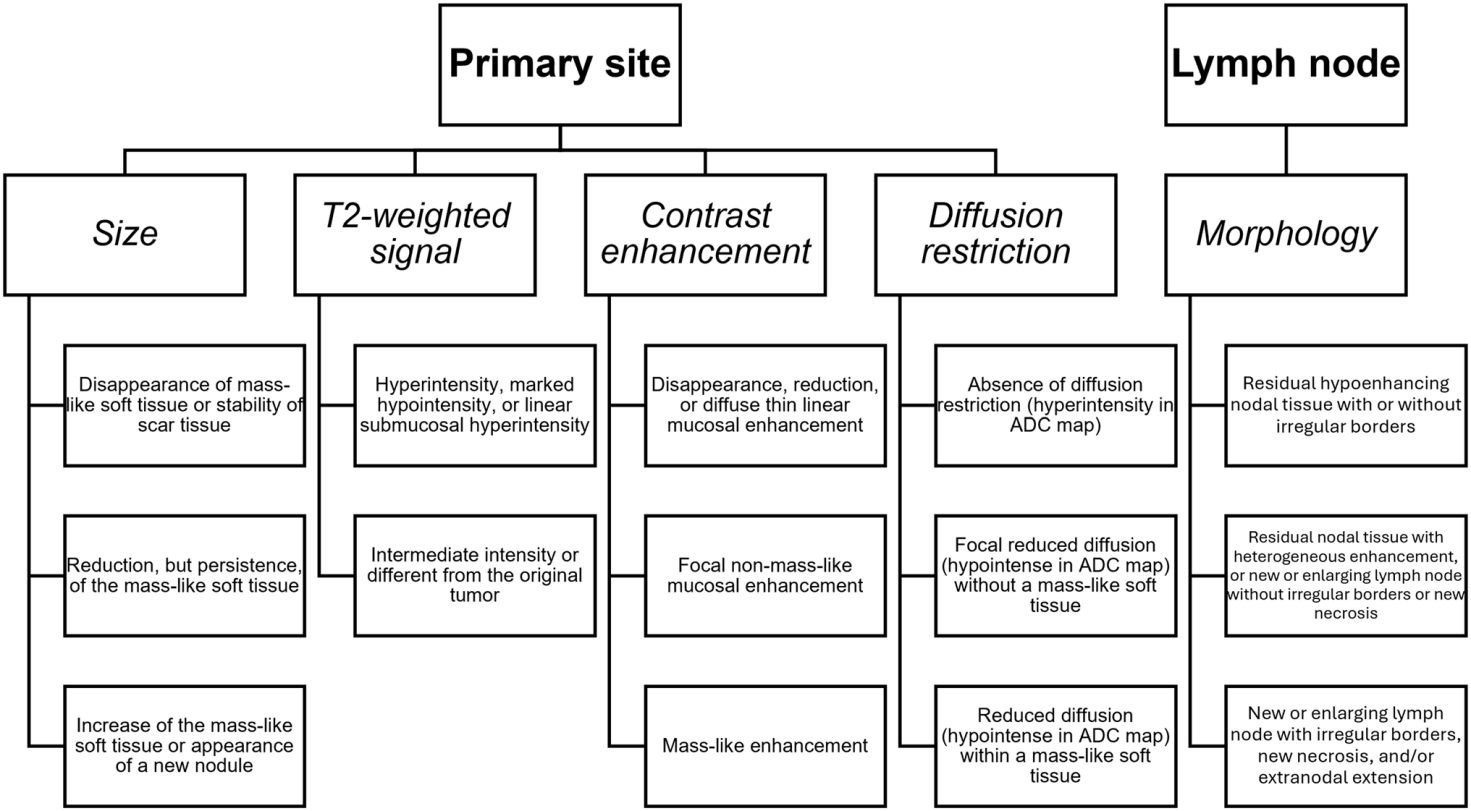
MRI NI-RADS features assessed by each reader: lymph node morphology, primary tumor size, contrast enhancement, and diffusion restriction were grouped into three categories, whereas T2-weighted signal at the primary site was divided into two. ADC, apparent diffusion coefficient

**Fig. 2:**
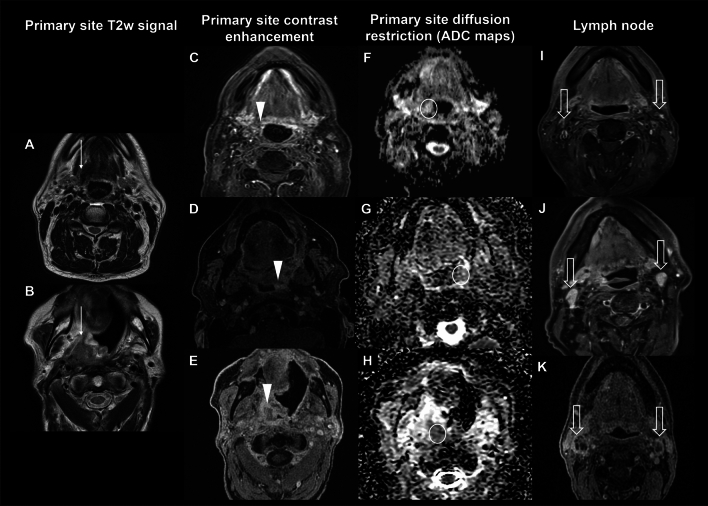
Examples of oropharyngeal carcinoma features after treatment. **A** Marked hypointene soft tissue and linear submucosal hyperintensity in the right tonsil (arrow), representing scar and edema. **B** Intermediate signal intensity (“evil grey”) soft tissue in the right tonsil (arrow), representing tumor recurrence. **C** Thin linear mucosal enhancement in the right tonsil (arrowhead), representing inflammation. **D** Focal non-mass-like mucosal enhancement in the left tonsil (arrowhead). **E** Mass-like enhancement in the right tonsil, representing tumor recurrence (arrowhead). **F** Absence of diffusion restriction (hyperintensity in ADC map) in the right tonsil (circle), representing edema. **G** Focal reduced diffusion (hypointense in ADC map) without a mass-like soft tissue in the left tonsil (circle). **H** Reduced diffusion (hypointense in ADC map) within a mass-like soft tissue in the right tonsil (circle), representing tumor recurrence. **I** Hypoenhancing nodal tissue without irregular borders (empty arrows) in a patient with base of the tongue carcinoma. **J** Residual nodal tissue with heterogeneous enhancement (empty arrows) in a patient with base of the tongue carcinoma. **K** Enlarging lymph node with irregular borders and new necrosis (empty arrows) in a patient with soft palate carcinoma. Some images are taken from the same patient exams (**A, C, F; B, E, H; D,G; I, J**). T2w, T2-weighted; ADC, apparent diffusion coefficient

The readers assigned a NI-RADS grade for each post-treatment CE-MRI scan, using the pre-treatment CE-MRI as reference for evaluating the first post-treatment CE-MRI. They also graded findings across the various MRI sequences according to the main NI-RADS features (Fig. 1). The readers referred to the November 2021 ACR NI-RADS descriptors, which were the current version available at the time of the study [20]. In cases where DWI was unavailable or T2-weighted imaging was compromised by artifacts, the assessment of those specific features was omitted. Nevertheless, the NI-RADS category was still determined based on the remaining diagnostic sequences. Figure 2 illustrates examples of the main CE-MRI features of OPSCC after treatment.

### Statistical analysis

To evaluate inter-observer reliability for NI-RADS and its CE-MRI features, we employed percentage of agreement (POA), Fleiss' kappa (κ) for five and three readers comparison, and Cohen's κ for pairwise comparison [21]. Inter-reader agreement was calculated for all post-treatment MRIs and separately for the first post-treatment CE-MRI and subsequent follow-ups.

Finally, to evaluate whether the NI-RADS score was influenced by the type of treatment or the location of the primary tumor, we conducted a one-way analysis of variance (ANOVA). The NI-RADS scores assigned by reader A at the first follow-up were grouped according to the treatment modality and anatomical site of the tumor.

## Results

Ninety-one patients were included in this cohort study, yielding 218 CE-MRI datasets for each rater and 1090 CE-MRI datasets for the analysis of inter-rater agreement (Tables 1 and 2). Inter-reader agreement is summarized as follows: Table 3 reports agreement across all post-treatment CE-MRIs; Table 4 reports agreement for the first post-treatment CE-MRI per patient; and Table 5 reports overall agreement for subsequent follow-up CE-MRIs (second and later). See supplementary information for the inter-observer agreement in the second, third, and fourth follow-up CE-MRIs, separately.

**Table 1:** Distribution of data included in the study

Variables	Values
Number of patients (male/female)	91 (67/24)
Mean age in years ± SD (range)	63 ± 8 (50–83)
Treatment (percentages):	
Radiotherapy Surgery Surgery plus chemoradiotherapy	59/91 (65%) 12/91 (13%) 20/91 (22%)
Oropharyngeal cancer location (percentages):	
Tonsil Base of tongue Soft palate Epiglottic vallecula Posterior pharyngeal wall	47/91 (52%) 30/91 (33%) 7/91 (8%) 4/91 (4%) 3/91 (3%)
Mean tumor volume at diagnosis in cubic centimeters ± SD (range)	19 ± 16 (0.42–76)
Number of patients based on MRI availability in follow ups (percentages):	
First follow up Second follow up Third follow up Fourth follow up	79/91 (87%) 73/91 (80%) 40/91 (44%) 26/91 (29%)
Mean time in days ± SD (range) between:	
Diagnosis and first follow up Treatment and first follow up Second and first follow up Third and second follow up Fourth and third follow up	203 ± 93 (46–420) 172 ± 93 (15–389) 257 ± 134 (69–549) 238 ± 96 (30–448) 282 ± 134 (59–504)
Number of datasets per reader (total number of datasets compared between 5 readers):	
MRI NI-RADS Primary tumor size Primary site T2-weighted signal Primary site contrast enhancement Primary site diffusion restriction on DWI Lymph node	218 (1090) 218 (1090) 218 (1090) 216 (1080) 218 (1090) 197 (985) 218 (1090)

SD, Standard deviation; DWI, Diffusion-weighted imaging; MRI, Magnetic resonance imaging; NI-RADS, Neck Imaging Reporting and Data System

**Table 2:** Relative frequency of the variables assigned by the five readers, with percentages in parentheses

Variables	Values
NI-RADS categories:	
1 2 3	839/1090 (77%) 117/1090 (11%) 134/1090 (12%)
Primary tumor size:	
Disappearance of mass-like soft tissue or stability of scar tissue Reduction, but persistence of the mass-like soft tissue I ncrease of the mass-like soft tissue or appearance of new nodule	903/1090 (83%) 99/1090 (9%) 88/1090 (8%)
Primary site T2-weighted signal:	
Hyperintensity, marked hypointensity, or linear submucosal hyperintensity Intermediate intensity or different from the original tumor	921/1080 (85%) 159/1080 (15%)
Primary site contrast enhancement:	
Disappearance, reduction or diffuse thin linear mucosal enhancement Focal non-mass-like mucosal enhancement Mass-like enhancement	887/1090 (81%) 57/1090 (5%) 146/1090 (14%)
Primary site diffusion restriction on DWI:	
Absence of diffusion restriction (hyperintensity in ADC map) Focal reduced diffusion (hypointense in ADC map) without a mass-like soft tissue Reduced diffusion (hypointense in ADC map) within a mass-like soft tissue	856/985 (87%) 23/985 (2%) 106/985 (11%)
Lymph node:	
Residual hypoenhancing nodal tissue with or without irregular borders Residual nodal tissue with heterogeneous enhancement; or new or enlarging lymph node without irregular borders or new necrosis New or enlarging lymph node with irregular borders, new necrosis and/or ENE	974/1090 (89%) 62/1090 (6%) 54/1090 (5%)

ADC, Apparent diffusion coefficient; DWI, Diffusion-weighted imaging; ENE, Extra nodal extension; NI-RADS, Neck Imaging Reporting and Data System

**Table 3:** Interrater agreement

	Variables	Kappa	Level of agreement according to kappa	Percentage of agreement
5 readers (A, B, C, D, E)	NI-RADS Primary tumor Size T2w signal Diffusion restriction Contrast enhancement Lymph node	0.53 [CI 95%: 0.51, 0.55] 0.52 [CI 95%: 0.50, 0.55] 0.52 [CI 95%: 0.49, 0.54] 0.51 [CI 95%: 0.48, 0.54] 0.47 [CI 95%: 0.45, 0.49] 0.42 [CI 95%: 0.39, 0.45]	Moderate Moderate Moderate Moderate Moderate Moderate	89% 92% 93% 84% 90% 93%
2 readers (A, B)	NI-RADS Primary tumor Size T2w signal Diffusion restriction Contrast enhancement Lymph node	0.64 [CI 95%: 0.52, 0.76] 0.52 [CI 95%: 0.36, 0.68] 0.60 [CI 95%: 0.45, 0.75] 0.54 [CI 95%: 0.36, 0.72] 0.57 [CI 95%: 0.43, 0.70] 0.65 [CI 95%: 0.44, 0.85]	Substantial Moderate Moderate Moderate Moderate Substantial	86% 86% 89% 89% 85% 95%
2 readers (A, C)	NI-RADS Primary tumor Size T2w signal Diffusion restriction Contrast enhancement Lymph node	0.51 [CI 95%: 0.37, 0.64] 0.48 [CI 95%: 0.32, 0.64] 0.39 [CI 95%: 0.19, 0.59] 0.34 [CI 95%: 0.13, 0.56] 0.43 [CI 95%: 0.27, 0.59] 0.36 [CI 95%: 0.13, 0.60]	Moderate Moderate Fair Fair Moderate Fair	81% 84% 86% 85% 83% 88%
2 readers (D, E)	NI-RADS Primary tumor Size T2w signal Diffusion restriction Contrast enhancement Lymph node	0.54 [CI 95%: 0.39, 0.68] 0.60 [CI 95%: 0.45, 0.74] 0.64 [CI 95%: 0.48, 0.79] 0.66 [CI 95%: 0.50, 0.82] 0.51 [CI 95%: 0.36, 0.67] 0.40 [CI 95%: 0.20, 0.60]	Moderate Moderate Substantial Substantial Moderate Fair	84% 88% 91% 92% 86% 86%
3 readers (A, D, E)	NI-RADS Primary tumor Size T2w signal Diffusion restriction Contrast enhancement Lymph node	0.54 [CI 95%: 0.50, 0.58] 0.57 [CI 95%: 0.53, 0.62] 0.65 [CI 95%: 0.60, 0.70] 0.62 [CI 95%: 0.56, 0.66] 0.52 [CI 95%: 0.47, 0.56] 0.40 [CI 95%: 0.35, 0.45]	Moderate Moderate Substantial Substantial Moderate Fair	92% 94% 96% 95% 93% 94%

Fleiss’ kappa is used for 5 and 3 readers reliability and Cohen’s kappa is used for 2 readers reliability. Percentage of agreement is the total number of cases in which all readers agree, divided by the total number of observations. A and B: expert head and neck radiologists; C: general radiologist; D and E: radiology residents; NI-RADS, Neck Imaging Reporting and Data System; T2w, T2-weighted; CI, confidence interval

**Table 4:** Interrater agreement at first follow up

	Variables	Kappa	Level of agreement according to kappa	Percentage of agreement
5 readers (A, B, C, D, E)	NI-RADS Primary tumor Size T2w signal Diffusion restriction Contrast enhancement Lymph node	0.49 [CI 95%: 0.47, 0.51] 0.43 [CI 95%: 0.41, 0.46] 0.45 [CI 95%: 0.41, 0.48] 0.48 [CI 95%: 0.44, 0.52] 0.40 [CI 95%: 0.37, 0.43] 0.37 [CI 95%: 0.33, 0.41]	Moderate Moderate Moderate Moderate Fair Fair	84% 76% 89% 90% 84% 88%
2 readers (A, B)	NI-RADS Primary tumor Size T2w signal Diffusion restriction Contrast enhancement Lymph node	0.52 [CI 95%: 0.34, 0.70] 0.41 [CI 95%: 0.17, 0.64] 0.51 [CI 95%: 0.28, 0.74] 0.47 [CI 95%: 0.22, 0.73] 0.50 [CI 95%: 0.31, 0.69] 0.63 [CI 95%: 0.37, 0.89]	Moderate Moderate Moderate Moderate Moderate Substantial	73% 76% 82% 82% 75% 91%
2 readers (A, C)	NI-RADS Primary tumor Size T2w signal Diffusion restriction Contrast enhancement Lymph node	0.51 [CI 95%: 0.33, 0.70] 0.45 [CI 95%: 0.24, 0.67] 0.35 [CI 95%: 0.10, 0.62] 0.31 [CI 95%: 0.01, 0.60] 0.38 [CI 95%: 0.15, 0.60] 0.25 [CI 95%: -0.01, 0.55]	Moderate Moderate Fair Fair Fair Fair	75% 76% 78% 77% 72% 77%
2 readers (D, E)	NI-RADS Primary tumor Size T2w signal Diffusion restriction Contrast enhancement Lymph node	0.48 [CI 95%: 0.27, 0.68] 0.45 [CI 95%: 0.23, 0.67] 0.54 [CI 95%: 0.28, 0.79] 0.58 [CI 95%: 0.33, 0.84] 0.41 [CI 95%: 0.16, 0.66] 0.35 [CI 95%: 0.09, 0.61]	Moderate Moderate Moderate Moderate Moderate Fair	76% 77% 86% 87% 78% 77%
3 readers (A, D, E)	NI-RADS Primary tumor Size T2w signal Diffusion restriction Contrast enhancement Lymph node	0.52 [CI 95%: 0.47, 0.56] 0.49 [CI 95%: 0.44, 0.54] 0.61 [CI 95%: 0.53, 0.68] 0.56 [CI 95%: 0.48, 0.63] 0.41 [CI 95%: 0.35, 0.47] 0.36 [CI 95%: 0.30, 0.43]	Moderate Moderate Substantial Moderate Moderate Fair	89% 89% 88% 92% 88% 90%

Fleiss’ kappa is used for 5 and 3 readers reliability and Cohen’s kappa is used for 2 readers reliability. Percentage of agreement is the total number of cases in which all readers agree, divided by the total number of observations. A and B: expert head and neck radiologists; C: general radiologist; D and E: radiology residents; NI-RADS, Neck Imaging Reporting and Data System; T2w, T2-weighted; CI, confidence interval

**Table 5:** Interrater agreement at second, third, and fourth follow up

	Variables	Kappa	Level of agreement according to kappa	Percentage of agreement
5 readers (A, B, C, D, E)	NI-RADS Primary tumor Size T2w signal Diffusion restriction Contrast enhancement Lymph node	0.53 [CI 95%: 0.50, 0.56] 0.59 [CI 95%: 0.55, 0.62] 0.57 [CI 95%: 0.53, 0.60] 0.53 [CI 95%: 0.49, 0.57] 0.52 [CI 95%: 0.48, 0.55] 0.44 [CI 95%: 0.40, 0.49]	Moderate Moderate Moderate Moderate Moderate Moderate	92% 95% 95% 95% 94% 96%
2 readers (A, B)	NI-RADS Primary tumor Size T2w signal Diffusion restriction Contrast enhancement Lymph node	0.73 [CI 95%: 0.57, 0.89] 0.61 [CI 95%: 0.40, 0.82] 0.67 [CI 95%: 0.47, 0.87] 0.60 [CI 95%: 0.35, 0.85] 0.61 [CI 95%: 0.41, 0.81] 0.65 [CI 95%: 0.32, 0.99]	Substantial Substantial Substantial Moderate Substantial Substantial	93% 91% 93% 93% 97% 93%
2 readers (A, C)	NI-RADS Primary tumor Size T2w signal Diffusion restriction Contrast enhancement Lymph node	0.46 [CI 95%: 0.25, 0.67] 0.45 [CI 95%: 0.19, 0.71] 0.41 [CI 95%: 0.11, 0.70] 0.37 [CI 95%: 0.06, 0.68] 0.45 [CI 95%: 0.20, 0.70] 0.51 [CI 95%: 0.16, 0.86]	Moderate Moderate Moderate Fair Moderate Moderate	85% 89% 90% 89% 88% 95%
2 readers (D, E)	NI-RADS Primary tumor Size T2w signal Diffusion restriction Contrast enhancement Lymph node	0.57 [CI 95%: 0.38, 0.77] 0.73 [CI 95%: 0.55, 0.91] 0.71 [CI 95%: 0.53, 0.91] 0.72 [CI 95%: 0.52, 0.92] 0.60 [CI 95%: 0.40, 0.80] 0.42 [CI 95%: 0.11, 0.73]	Moderate Substantial Substantial Substantial Moderate Moderate	88% 94% 94% 94% 90% 91%
3 readers (A, D, E)	NI-RADS Primary tumor Size T2w signal Diffusion restriction Contrast enhancement Lymph node	0.53 [CI 95%: 0.47, 0.59] 0.63 [CI 95%: 0.56, 0.70] 0.68 [CI 95%: 0.61, 0.75] 0.65 [CI 95%: 0.58, 0.73] 0.60 [CI 95%: 0.54, 0.67] 0.42 [CI 95%: 0.34, 0.49]	Moderate Substantial Substantial Substantial Moderate Moderate	94% 96% 97% 97% 95% 96%

Fleiss’ kappa is used for 5 and 3 readers reliability and Cohen’s kappa is used for 2 readers reliability. Percentage of agreement is the total number of cases in which all readers agree, divided by the total number of observations. A and B: expert head and neck radiologists; C: general radiologist; D and E: radiology residents; NI-RADS, Neck Imaging Reporting and Data System; T2w, T2-weighted; CI, confidence interval

The same NI-RADS grades were assigned by all radiologists in 145 out of 218 cases (67%). A consensus was reached by four readers in 31 out of 218 cases (14%), while three radiologists agreed on 39 out of 218 cases (18%). Only two readers concurred on the NI-RADS assignment in 3 out of 218 cases (1%).

The agreement for NI-RADS (κ = 0.53, POA = 89%) and its individual features (κ = 0.42–0.52, POA = 84–93%) was moderate among all readers, with the lowest κ value for lymph node evaluation. When examining lymph nodes in the subgroup analysis, fair agreement was observed between reader A and reader C (κ = 0.36, POA = 88%), as well as among readers A, D and E (κ = 0.40, POA = 94%).

In the first follow-up, the agreement for NI-RADS was moderate (κ = 0.49, POA = 84%), while it was fair for lymph node evaluation (κ = 0.37, POA = 88%) and primary site contrast enhancement (κ = 0.40, POA = 84%). Specifically, the analysis confirmed low reliability in lymph node evaluation and primary site contrast enhancement between reader A and reader C (κ = 0.25, POA = 77% and κ = 0.38, POA = 72%, respectively). Low reliability was also found between the readers A, D and E for these features (κ = 0.36, POA = 90% and κ = 0.41, POA = 88%, respectively).

The agreement for NI-RADS (κ = 0.53, POA = 92%) and its individual features (κ = 0.44–0.59, POA = 94%-96%) was moderate across the second, third, and fourth follow-up MRI scans, with lymph node evaluation showing the lowest κ value. Furthermore, the reliability for assessing primary site contrast enhancement and lymph nodes improved in the later follow-up scans compared to the initial one. This increase was observed both in the agreement between the readers A, D and E (for nodal evaluation κ = 0.42, POA = 96%; for primary site contrast enhancement κ = 0.60, POA = 95%) and between the reader A and C (for nodal evaluation κ = 0.51, POA = 95%; for primary site contrast enhancement κ = 0.45, POA = 88%).

**Fig. 3:**
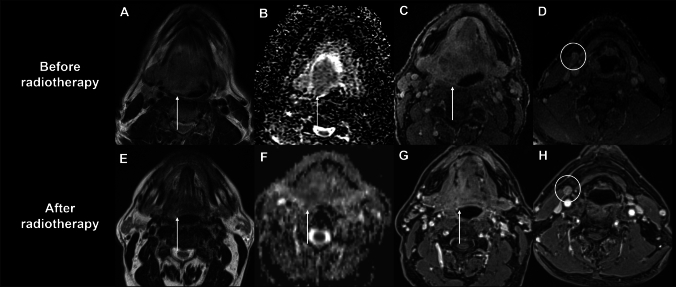
An example of low agreement in the evaluation of an oropharyngeal squamous cell carcinoma (OPSCC) of the base of the tongue on the first post-radiotherapy magnetic resonance imaging. **A** and E. T2-weighted images; **B** and **F.** Apparent diffusion coefficient maps; **C**, **D**, **G** and **H**. T1-weighted images after contrast agent administration. **A**, **B**, **C** and **D** show a large OPSCC (arrows) with enlarged cervical lymph nodes (circle) before treatment. **E**, **F**, **G** and **H** show partial resolution of OPSCC with persistent enlarged cervical lymph node (circle) after treatment. There was variable disagreement among all readers in classifying the individual NI-RADS features (lymph node status, primary tumor size, and primary site signal on T2-weighted, contrast-enhanced, and diffusion-weighted images). The final NI-RADS category assigned was 1 for the general radiologist, 2 for the expert radiologists and a radiology resident, and 3 for a radiology resident

Figure 3 illustrates a case of limited concordance among radiologists in the assessment of the first post-radiation CE-MRI.

The ANOVA results showed no statistically significant differences in NI-RADS scores assigned by reader A at the first follow-up across different treatment groups (*p* = 0.78) or tumor locations (*p* = 0.30).

## Discussion

The findings of our study demonstrated a moderate degree of consistency in NI-RADS evaluations on CE-MRI among readers with different levels of radiological expertise. We also observed moderate inter-rater agreement for each specific CE-MRI feature of NI-RADS that was analyzed, including primary tumor size, T2-weighted signal intensity, contrast enhancement, diffusion restriction at the primary site, and lymph node characteristics. The results indicated that reader expertise influenced the outcomes: subgroup analysis showed that radiologists with less specialization in head and neck imaging, namely the general radiologist and the radiology residents, demonstrated lower levels of agreement compared to their more experienced counterparts. Furthermore, aligning with the NI-RADS framework, which distinguishes between the first imaging assessment after treatment and subsequent follow-up scans, we performed a separate sub-analysis of CE-MRIs. The consistency in evaluating lymph nodes and primary site contrast enhancement was lower in the initial follow-up CE-MRI scans compared to later ones, particularly within the subgroups that included radiologists without specific head and neck expertise. These findings imply that the initial post-treatment CE-MRI presents greater interpretative challenges for radiologists. This may lead to conservative categorization, particularly when classifying nodal residual tissue as predominantly hypoenhancing versus heterogeneously enhancing, and when differentiating expected treatment-related primary-site enhancement (e.g., diffuse thin linear or focal mucosal enhancement) from a mass-like enhancement pattern that is more suspicious for residual tumor.

When interpreting our results, it is important to consider that the observed difference between the POA and the κ coefficient underscores a known weakness of the κ statistic in analyzing imbalanced data. For example, the assessment of lymph nodes by readers A and C showed substantial divergence: a low κ value of 0.36 contrasted with a high POA of 88%. This suggests that the seemingly high agreement might be inflated due to the dominance of one diagnostic category, where the probability of chance agreement increases [22, 23].

As far as we know, no prior studies have focused on inter-reader reliability for CE-MRI NI-RADS within a cohort of radiologists with diverse backgrounds in OPSCC assessment. This gap in literature presents a challenge for direct comparison, and any differences between our findings and previous work likely stem from variations in the populations examined, imaging methodologies, and the experience of the interpreting radiologists. In the work by Elsholtz et al., three seasoned head and neck radiologists independently evaluated 104 head and neck cancer patients (including 25 with OPSCC) using CE-MRI. They found a moderate inter-reader agreement (Fleiss' κ = 0.53) for the primary site assessment according to NI-RADS, whereas lymph node evaluation showed substantial agreement (Fleiss' κ = 0.67). For DWI evaluation of the primary site, excellent consistency was observed (Fleiss' κ = 0.83) when selecting between the presence of clear diffusion restriction and the absence or ambiguity of diffusion restriction [24]. In another study by Elsholtz et al., four radiologists with differing levels of experience evaluated CE-CT scans from 101 patients, including 29 with OPSCC. NI-RADS showed a moderate agreement for both the primary site (Fleiss' κ = 0.48) and the lymph nodes (Fleiss' κ = 0.50) [25]. Abdelaziz et al. reported substantial concordance in evaluating the primary tumor site (κ = 0.78, POA = 85%) with CE-MRI in a cohort of carcinomas without OPSCC. Moreover, lymph node evaluation showed almost perfect agreement (κ = 0.85, POA = 91%) [26]. It is interesting to highlight that the readers in the current study have already conducted a similar interobserver agreement study on 30 patients (for a total of 94 MRI scans analyzed per reader) with nasopharyngeal carcinoma. In that study, the radiologists assigned the NI-RADS and evaluated individual CE-MRI features, not strictly following the description in the original NI-RADS table, but in a more simplistic manner in terms of stability, reduction, or increase. Even in that case a greater difficulty was found in evaluating the first CE-MRI after treatment, particularly regarding primary site contrast enhancement. Moreover, a moderate agreement was found in assigning the NI-RADS (κ = 0.41 vs. κ = 0.53), but the POA values were generally lower than those found in the current study (POA = 65–87% vs. POA = 84–93%), although with a higher κ in the evaluation of lymph nodes (κ = 0.68 vs. κ = 0.42) [19]. Two main points may account for the discrepancies between studies: first, a learning curve exists for all readers when using a new scoring system, as shown by the improved POA after a systematic training set of 94 nasopharyngeal cancer cases; second, it is likely that applying NI-RADS criteria to CE-MRI features is inherently complex, given the lower inter-reader agreement found when evaluating nodal margins and enhancement patterns in addition to size. The recent publication of the updated ACR NI-RADS table for CE-MRI (August 2025) [27], alongside ACR-supported practical guidelines [28], underscores the strong and ongoing interest in this RADS framework. We believe that the implementation of further educational tools on the official NI-RADS webpage [12] will lead to a continued improvement in NI-RADS reliability within daily clinical practice.

It is important to consider some limitations intrinsic to this study. We included examinations from different MRI scanners within a considerable timeframe, introducing a source of potential inconsistencies in image acquisition. In a limited number of instances, DWI sequences were missing because standardized head and neck imaging protocols were not in place during earlier examinations. However, to enroll the largest possible number of OPSCC patients with at least two consecutive CE-MRI scans, a long data collection period was necessary. The retrospective design limited our ability to fully access patient clinical data for correlation analysis. However, the diagnostic performance of NI-RADS has been extensively studied and recently summarized in a meta-analysis [15]. Finally, the relatively high occurrence of NI-RADS 1 scores, indicating a low number of complex presentations, might have made the assessment process simpler, potentially affecting the κ values. Nevertheless, this distribution is representative of the general prevalence of imaging findings encountered in clinical practice.

## Conclusion

The inter-rater reliability of MRI NI-RADS in the surveillance of OPSCC patients is moderate among readers with varying expertise. Greater uncertainty is evident in the evaluation of primary site contrast enhancement and lymph nodes at the first follow-up for radiologists who are not sub-specialized in head and neck imaging. Routinely applying the NI-RADS scoring system can enhance reliability, strengthen readers' confidence in assigning categories, and ultimately support more informed clinical decision-making.

## Supplementary Information

Below is the link to the electronic supplementary material.

Supplementary file 1 (PDF 152 KB)
