# Supplementary material for: Inter-rater reliability of MRI Neck Imaging Reporting and Data System (NI-RADS) in the follow-up of oropharyngeal squamous cell carcinoma
Source: Radiol Med. 2026 Mar 30;131(7):1237–47. doi: 10.1007/s11547-026-02206-z (PMC13369041; doi:10.1007/s11547-026-02206-z)
Supplement: Supplementary file 1 — (PDF 152 KB) [file 11547_2026_2206_MOESM1_ESM.pdf]

Table 6 Interrater agreement at second follow up. Fleiss' kappa is used for 5 and 3 readers reliability and Cohen's kappa is used for 2 readers reliability. Percentage of agreement is the total number of cases in which all readers agree, divided by the total number of observations. A and B: expert head and neck radiologists; C: general radiologist; D and E: radiology residents; NI-RADS, Neck Imaging Reporting and Data System; T2w, T2-weighted; CI, confidence interval.

	**Variables**	**Kappa**	**Level of agreement according to kappa**	**Percentage of agreement**
5 readers (A, B, C, D, E)	NI-RADS	0.47 [CI 95%: 0.33, 0.60]	Moderate	89%
	Primary tumor			
	Size	0.61 [CI 95%: 0.48, 0.74]	Substantial	93%
	T2w signal	0.59 [CI 95%: 0.44, 0.74]	Moderate	94%
	Diffusion restriction	0.52 [CI 95%: 0.34, 0.69]	Moderate	93%
	Contrast enhancement	0.53 [CI 95%: 0.39, 0.67]	Moderate	92%
	Lymph node	0.17 [CI 95%: -0.19, 0.52]	Slight	94%
2 readers (A, B)	NI-RADS	0.62 [CI 95%: 0.39, 0.85]	Substantial	88%
	Primary tumor			
	Size	0.62 [CI 95%: 0.37, 0.87]	Substantial	89%
	T2w signal	0.64 [CI 95%: 0.39, 0.89]	Substantial	90%
	Diffusion restriction	0.53 [CI 95%: 0.20, 0.86]	Moderate	89%
	Contrast enhancement	0.59 [CI 95%: 0.34, 0.84]	Moderate	88%
	Lymph node	0.49 [CI 95%: -0.21, 1]	Moderate	97%
2 readers (A, C)	NI-RADS	0.37 [CI 95%: 0.11, 0.64]	Fair	77%
	Primary tumor			
	Size	0.46 [CI 95%: 0.16, 0.75]	Moderate	85%
	T2w signal	0.40 [CI 95%: 0.03, 0.77]	Fair	88%
	Diffusion restriction	0.24 [CI 95%: -0.17, 0.65]	Fair	83%
	Contrast enhancement	0.51 [CI 95%: 0.22, 0.79]	Moderate	86%
	Lymph node	0.23 [CI 95%: -0.37, 0.82]	Fair	92%
2 readers (D, E)	NI-RADS	0.51 [CI 95%: 0.25, 0.76]	Moderate	84%
	Primary tumor			
	Size	0.75 [CI 95%: 0.54, 0.96]	Substantial	93%
	T2w signal	0.68 [CI 95%: 0.43, 0.92]	Substantial	92%
	Diffusion restriction	0.70 [CI 95%: 0.44, 0.95]	Substantial	92%
	Contrast enhancement	0.53 [CI 95%: 0.28, 0.79]	Moderate	85%
	Lymph node	0.04 [CI 95%: -0.51, 0.59]	Slight	86%
3 readers (A, D, E)	NI-RADS	0.48 [CI 95%: 0.31, 0.66]	Moderate	92%
	Primary tumor			
	Size	0.67 [CI 95%: 0.54, 0.81]	Substantial	95%
	T2w signal	0.74 [CI 95%: 0.63, 0.84]	Substantial	97%
	Diffusion restriction	0.72 [CI 95%: 0.61, 0.84]	Substantial	96%
	Contrast enhancement	0.63 [CI 95%: 0.50, 0.77]	Substantial	94%
	Lymph node	0.11 [CI 95%: -0.32, 0.55]	Slight	94%

Table 7 Interrater agreement at third follow up. Fleiss' kappa is used for 5 and 3 readers reliability and Cohen's kappa is used for 2 readers reliability. Percentage of agreement is the total number of cases in which all readers agree, divided by the total number of observations. A and B: expert head and neck radiologists; C: general radiologist; D and E: radiology residents; NI-RADS, Neck Imaging Reporting and Data System; T2w, T2-weighted; CI, confidence interval.

	**Variables**	**Kappa**	**Level of agreement according to kappa**	**Percentage of agreement**
5 readers (A, B, C, D, E)	NI-RADS	0.64 [CI 95%: 0.45, 0.83]	Substantial	95%
	Primary tumor			
	Size	0.60 [CI 95%: 0.35, 0.85]	Moderate	97%
	T2w signal	0.59 [CI 95%: 0.35, 0.83]	Moderate	96%
	Diffusion restriction	0.66 [CI 95%: 0.41, 0.90]	Substantial	97%
	Contrast enhancement	0.52 [CI 95%: 0.26, 0.78]	Moderate	95%
	Lymph node	0.79 [CI 95%: 0.62, 0.95]	Substantial	98%
2 readers (A, B)	NI-RADS	0.89 [CI 95%: 0.69, 1]	Almost perfect	97%
	Primary tumor			
	Size	0.72 [CI 95%: 0.35, 1]	Substantial	95%
	T2w signal	0.72 [CI 95%: 0.35, 1]	Substantial	95%
	Diffusion restriction	0.79 [CI 95%: 0.38, 1]	Substantial	97%
	Contrast enhancement	0.75 [CI 95%: 0.42, 1]	Substantial	95%
	Lymph node	0.79 [CI 95%: 0.38, 1]	Substantial	97%
2 readers (A, C)	NI-RADS	0.63 [CI 95%: 0.23, 1]	Substantial	92%
	Primary tumor			
	Size	0.47 [CI 95%: -0.10, 1]	Moderate	92%
	T2w signal	0.44 [CI 95%: -0.07, 0.96]	Fair	90%
	Diffusion restriction	0.82 [CI 95%: 0.48, 1]	Almost perfect	97%
	Contrast enhancement	0.39 [CI 95%: -0.17, 0.96]	Fair	90%
	Lymph node	0.79 [CI 95%: 0.37, 1]	Substantial	97%
2 readers (D, E)	NI-RADS	0.67 [CI 95%: 0.30, 1]	Substantial	92%
	Primary tumor			
	Size	0.69 [CI 95%: 0.28, 1]	Substantial	95%
	T2w signal	0.84 [CI 95%: 0.54, 1]	Almost perfect	97%
	Diffusion restriction	0.82 [CI 95%: 0.47, 1]	Almost perfect	97%
	Contrast enhancement	0.72 [CI 95%: 0.35, 1]	Substantial	95%
	Lymph node	0.85 [CI 95%: 0.55, 1]	Almost perfect	97%
3 readers (A, D, E)	NI-RADS	0.63 [CI 95%: 0.41, 0.84]	Substantial	96%
	Primary tumor			
	Size	0.65 [CI 95%: 0.43, 0.88]	Substantial	97%
	T2w signal	0.70 [CI 95%: 0.51, 0.89]	Substantial	98%
	Diffusion restriction	0.64 [CI 95%: 0.39, 0.90]	Substantial	97%
	Contrast enhancement	0.63 [CI 95%: 0.39, 0.86]	Substantial	97%
	Lymph node	0.76 [CI 95%: 0.65, 0.88]	Slight	98%

Table 8 Interrater agreement at fourth follow up. Fleiss' kappa is used for 5 and 3 readers reliability and Cohen's kappa is used for 2 readers reliability. Percentage of agreement is the total number of cases in which all readers agree, divided by the total number of observations. A and B: expert head and neck radiologists; C: general radiologist; D and E: radiology residents; NI-RADS, Neck Imaging Reporting and Data System; T2w, T2-weighted; CI, confidence interval.

	**Variables**	**Kappa**	**Level of agreement according to kappa**	**Percentage of agreement**
5 readers (A, B, C, D, E)	NI-RADS	0.62 [CI 95%: 0.28, 0.95]	Substantial	97%
	Primary tumor			
	Size	0.12 [CI 95%: -0.63, 0.87]	Slight	96%
	T2w signal	0.21 [CI 95%: -0.43, 0.86]	Fair	95%
	Diffusion restriction	0.32 [CI 95%: -0.23, 0.87]	Fair	95%
	Contrast enhancement	0.34 [CI 95%: -0.16, 0.84]	Fair	95%
	Lymph node	0.54 [CI 95%: 0.14, 0.94]	Moderate	97%
2 readers (A, B)	NI-RADS	1 [CI 95%: 1, 1]	Perfect	100%
	Primary tumor			
	Size	0	-	92%
	T2w signal	0.65 [CI 95%: -0.01, 1]	Substantial	96%
	Diffusion restriction	0.65 [CI 95%: -0.01, 1]	Substantial	96%
	Contrast enhancement	0.31 [CI 95%: -0.62, 1]	Fair	92%
	Lymph node	0.65 [CI 95%: -0.03, 1]	Substantial	96%
2 readers (A, C)	NI-RADS	0.65 [CI 95%: -0.01, 1]	Substantial	96%
	Primary tumor			
	Size	0	-	96%
	T2w signal	0	-	96%
	Diffusion restriction	0	-	92%
	Contrast enhancement	0	-	92%
	Lymph node	1 [CI 95%: 1, 1]	Perfect	100%
2 readers (D, E)	NI-RADS	0.73 [CI 95%: 0.22, 1]	Substantial	96%
	Primary tumor			
	Size	0.65 [CI 95%: -0.03, 1]	Substantial	96%
	T2w signal	0.65 [CI 95%: -0.03, 1]	Substantial	96%
	Diffusion restriction	0.65 [CI 95%: -0.03, 1]	Substantial	96%
	Contrast enhancement	0.74 [CI 95%: 0.22, 1]	Substantial	96%
	Lymph node	0.74 [CI 95%: 0.22, 1]	Substantial	96%
3 readers (A, D, E)	NI-RADS	0.55 [CI 95%: 0.17, 0.94]	Moderate	96%
	Primary tumor			
	Size	0.22 [CI 95%: -0.51, 0.94]	Fair	96%
	T2w signal	0.21 [CI 95%: -0.52, 0.94]	Fair	96%
	Diffusion restriction	0.21 [CI 95%: -0.52, 0.94]	Fair	96%
	Contrast enhancement	0.26 [CI 95%: -0.36, 0.88]	Fair	95%
	Lymph node	0.58 [CI 95%: 0.18, 0.97]	Moderate	97%
